# Lunasin is a novel therapeutic agent for targeting melanoma cancer stem cells

**DOI:** 10.18632/oncotarget.11554

**Published:** 2016-08-23

**Authors:** Chris Shidal, Numan Al-Rayyan, Kavitha Yaddanapudi, Keith R. Davis

**Affiliations:** ^1^ Department of Pharmacology and Toxciology, University of Louisville School of Medicine, Louisville, Kentucky, USA; ^2^ Department of Medicine, University of Louisville School of Medicine, Louisville, Kentucky, USA; ^3^ James Graham Brown Cancer Center, University of Louisville School of Medicine, Louisville, Kentucky, USA; ^4^ Biotechnology Program, Indiana University, Bloomington, Indiana, USA

**Keywords:** lunasin, melanoma, cancer stem cells, MITF, NANOG

## Abstract

Recent studies provide compelling evidence that melanoma is initiated and maintained by a small population of malignant cells called cancer-initiating cells (CICs) that exhibit stem-cell-like properties. Observations that CICs have a distinct biology when compared to that of the bulk tumor cells and, importantly, are resistant to chemotherapies and radiation, suggest that CICs are involved in invasion, metastasis, and ultimately relapse. Lunasin, a bioactive peptide present in soybean, has both chemopreventive activity and chemotherapeutic activity against multiple cancer types. In this study, we tested the potential of Lunasin to specifically target CICs in melanoma tumor cell populations. *In vitro* studies using human melanoma cell lines showed that Lunasin treatment decreased the size of a subpopulation of melanoma cells expressing the surrogate CIC marker, Aldehyde Dehydrogenase, concomitant with a reduction in the ability to form colonies in soft agar assays, and reduced tumor growth in mouse xenografts. Similarly, Lunasin inhibited colony formation by isolated melanoma CICs in soft agar and reduced oncosphere formation *in vitro* and substantially inhibited tumor growth in mouse xenografts. Mechanistic studies revealed that Lunasin treatment of isolated melanoma CICs induced expression of the melanocyte-associated differentiation markers Tyrosinase and Microphthalmia-associated Transcription Factor concomitant with reduced expression of the stemness factor NANOG. These findings document for the first time that Lunasin has significant therapeutic activity against melanoma by specifically targeting melanoma CICs, and inducing a more differentiated, non-CIC phenotype. Thus, Lunasin may represent a novel therapeutic option for both chemoresistant and advanced metastatic melanoma management.

## INTRODUCTION

Skin cancers account for nearly half of all diagnosed cancer cases in the United States and have increased in frequency over the last thirty years [1]. Despite being less frequent than other skin cancers, nearly 75% of skin cancer deaths are attributed to melanoma. NCI's Surveillance, Epidemiology, and End Results program estimates that cases of melanoma have nearly tripled in the past thirty years; the number of cases has increased from 7.9 (per 100,000) in 1975 to 22.7 in 2011, while the 5-year survival rate remains constant [2]. Recurrent disease is the major cause of morbidity and mortality associated with melanoma. Although significant progress has been made in preventing or delaying disease, additional non-toxic approaches are needed to reduce the risk of recurrence. Studies in preclinical models of carcinogenesis have shown that an enrichment of melanoma cancer initiating cells (CICs) is likely to occur after conventional chemotherapeutic regimens, implicating CICs in treatment resistance and cancer recurrence [3–6]. Thus, successful elimination of CICs, along with the proliferating bulk tumor melanoma cells could be an effective therapeutic strategy to achieve higher rates of complete remission, especially in patients with late stage melanoma.

Melanoma CICs have been shown to represent about 1–25% of all tumor cells and can form tumors by injection of a single cell [7]. Identification of a “universal” biomarker for CICs remains a major research focus [8]; however, most markers appear to be model specific. Melanoma cells with stem-cell-like plasticity were initially discovered in patient tumors that overexpressed CD20 [9] and CD133 [10]. These subsets of cells displayed characteristics of stem cells and exhibited an enhanced ability to form palpable tumors in immunodeficient mice. Ensuing studies have identified ABCB5 [11] and CD271 [12] as potential melanoma CIC biomarkers. More recently, melanoma cells expressing Aldehyde Dehydrogenase (ALDH) have been shown to display stem-cell-like properties with enhanced *in vivo* tumorigenic capacity [13]. Other studies utilizing solid tumor models of the colon [14], breast [15], and lung [16] provide further evidence for utilizing expression levels of ALDH as a CIC marker. This hypothesis is supported by data showing ALDH1 expression correlates with poor prognosis in breast [17], ovarian [18], and lung [19] cancers, and that ALDH is critical in the development and differentiation of hematopoietic stem cells [20, 21] by modulating retinoid signaling through the conversion of vitamin A (retinol) to retinoic acid [22], a ligand for downstream nuclear receptors retinoic acid receptor (RAR) and retinoid X receptor (RXR) [23].

Lunasin, a 43–44 amino acid peptide component of the 2S albumin protein, has three putative functional domains including an aspartic acid tail, an RGD domain, and a chromatin-binding helical domain [24, 25]. Lunasin has been shown to exhibit robust chemopreventive and chemotherapeutic activities [26–30]. Lunasin has chemotherapeutic activity both *in vitro* and *in vivo* in various cancer models, including colon [31–33] and breast [34] cancer. Previous studies from our laboratory have established a novel, functional role for Lunasin in decreasing proliferation of non-small cell lung cancer (NSCLC) cells by suppressing integrin signaling through α_v_β_3_ [35, 36]. This finding is consistent with results from previous studies that demonstrate that Lunasin is internalized via α_v_β_3_ integrin [26, 37]. When compared to melanoma cells, the expression of α_v_β_3_ integrins are lower in non-transformed epithelial cells [38]; the expression levels of α_v_β_3_ correlate with the metastatic potential and the conversion of melanoma neoplasms to a metastatic phenotype [39].

In light of recent studies that clearly link integrin-matrix interactions to cancer cell survival [40], including the maintenance and survival of CICs through integrin-FAK signaling [41–49], we asked whether Lunasin can target melanoma CICs and, if yes, is this anti-CIC activity critical for its *in vivo* anti-tumorigenic effects. Our results show, for the first time, that Lunasin specifically targets ALDH^high^ CICs in human melanoma cell lines. Lunasin treatment decreases the expression of surrogate CIC markers *in vitro* and affects their *in vivo* tumorigenicity. Lunasin treatment significantly reduces formation of CIC-enriched melanoma oncospheres and, more importantly, induces expression of melanocyte-associated differentiation markers while suppressing a stem-cell-associated factor. Taken together, our results delineate the ability of Lunasin to regulate melanoma CIC properties and provide a compelling argument for developing Lunasin as a therapeutic agent to reduce melanoma recurrence.

## RESULTS

### Lunasin inhibits anchorage-independent growth in human melanoma cell lines

**Figure 1:**
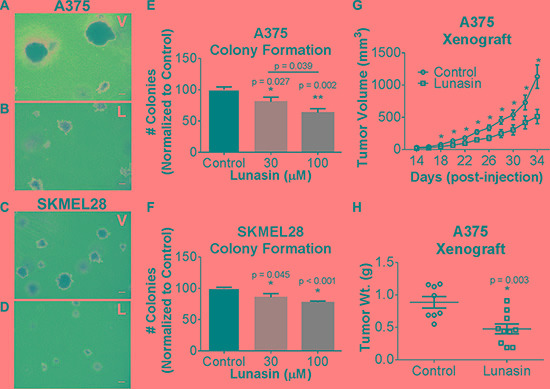
*In vitro* efficacy of lunasin in malignant melanomas Representative images of colonies grown in soft agar for vehicle-treated (**A**, **C**) and Lunasin-treated (**B**, **D**) A375 (top panels) and SKMEL-28 (bottom panels) cells (magnification at 40×). Scale bars on images represent 100 μm. Anchorage-independent growth conditions sensitized melanoma cells to Lunasin resulting in a significant decrease in colony formation in A375 (**E**) and SKMEL-28 (**F**) cells. Statistical significance between treatment groups is denoted by a different number of asterisks (*, **) and *p*-values are provided for each significant difference. Error bars on graphs represent mean ± S.D. For xenograft studies (2.5 × 10^6^ A375 cells were injected s.c. into nude mice and subsequently treated with vehicle (*n* = 8) or Lunasin (*n* = 10) for a total of 34 d. Lunasin reduced tumor volume by 55% (**G**) and wet tumor weight by 46% (**H**). Lunasin-treated mice differed significantly in tumor volume (*p* < 0.001) from control treated mice. The corresponding reduction in wet tumor weights were determined to be significant by unpaired student's *t-test* (*p* = 0.003) and denoted by an asterisk (*). Error bars represent mean ± S.E.M.

Our previous studies of NSCLC demonstrated that Lunasin had modest or no effect on most cell lines when grown under standard adherent culture conditions whereas all cell lines tested were sensitive under non-adherent conditions over a dose range of 10 to 100 μM [36]. We found that Lunasin was also effective over this dose range with human melanoma cell lines. A375 and SKMEL-28 cells did not show any decrease in proliferation in adherent culture when treated with a concentration range of 10 to 100 μM over three days when assayed using a standard 3-(4,5-dimethylthiazol-2-yl)-5-(3-carboxymethoxyphenyl)-2-(4-sulfophenyl)-2H-tetrazolium (MTS) based assay (data not shown). However, A375 and SKMEL-28 melanoma cells exhibited a significant dose-dependent decrease in colony formation in soft agar assays upon exposure to Lunasin (Figure 1D and 1E). When compared to cells treated with vehicle alone; colony formation by A375 cells was reduced 37% upon treatment with 100 μM Lunasin (Figure 1A, 1B, and 1E), while Lunasin-treated SKMEL-28 cells exhibited a 23% inhibition of colony formation (Figure 1C–1F). The size of colonies formed by cells was also reduced upon exposure to Lunasin (Figure 1A–1D). These results establish that Lunasin inhibits anchorage-independent growth of melanoma *in vitro* and provides the first demonstration that Lunasin has therapeutic effects on human melanoma cells.

### Lunasin inhibits tumor growth of melanoma cells *in vivo*

To evaluate whether the inhibition of *in vitro* anchorage-independent growth of melanoma cells can be recapitulated *in vivo*, we established tumor xenografts by subcutaneous (s.c.) implantation of A375 cells in athymic nude mice. Tumor cell implantation was followed by concurrent and subsequent daily intraperitoneal (i.p.) injections of Lunasin (30 mg/kg body weight). Dosing was determined by a combination of our previous preliminary *in vivo* experiments (data not shown) with the objective of obtaining a significant host response without obvious side effects or toxicity, and established dosing regimens for previously established biologics (e.g. cilengitide). While we did not observe a significant reduction in palpable tumor initiation, a significant reduction in the tumor growth rate was observed in the Lunasin-treated mice when compared to mice injected with vehicle alone (Figure 1G). Measurement of tumors began 14 days after initial implantation; however, we observed that mice in the Lunasin-treated group displayed tumors at this time that were significantly smaller (< 50 mm^3^) and difficult to measure due to their small size and lack of depth. Mice treated with Lunasin over a 34 day period exhibited significant reductions in tumor volume (55%) and total tumor mass (46%) when compared to those measured in the vehicle-treated mice (Figure 1G and 1H). These results established that Lunasin was biologically active *in vivo* in this mouse model.

### Lunasin reduces the melanoma CIC subpopulation in established cell lines

**Figure 2:**
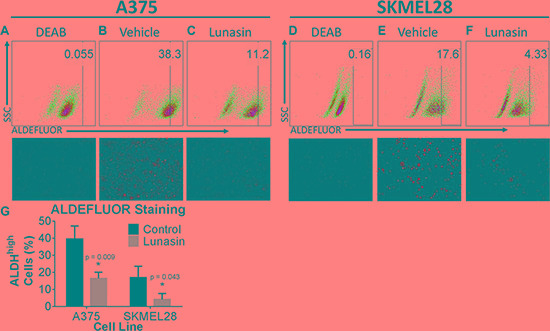
Lunasin depleted populations of cells displaying high ALDH activity A375 and SKMEL-28 cells showed a substantial decrease in ALDH positive populations when treated with Lunasin for 24 h. DEAB was used as a negative control and served as a tool for gating ALDH negative populations; the gating was kept constant for each cell line and is indicated within each plot. Representative flow cytometry plots and corresponding fluorescent microscopy images were taken at 24 h post-treatment and provided for DEAB (**A**, **D**), control (**B**,**E**), and Lunasin-treated (**C**, **F**) groups. Lunasin reduced the number ALDH^high^ cells in A375 and SKMEL-28 cell lines when compared to vehicle-treated cells (**G**). Statistical significance was determined from three independent experiments and assessed by student *t-test* (*p* < 0.05). Fluorescent microscopy images were taken at 40× magnification. Error bars represent mean ± S.D.

ALDH is an intracellular enzyme highly expressed by stem-cell-like cells [50] and recent studies suggest that high ALDH activity is a property of human melanoma CICs [13]. To measure ALDH activity in melanoma cells, we employed ALDEFLUOR, a commercially available molecule that freely diffuses into cells and is a substrate for the ALDH enzyme. ALDH cleaves ALDEFLUOR and yields a fluorescent product that can no longer diffuse across the cell membrane. ALDH activity within the cells is then assayed by incubating cells with a fluorescent ALDH substrate followed by flow cytometry. For this assay, ALDH^high^ CICs were identified by comparing the fluorescence in a test sample to that in a control sample containing DEAB, a specific inhibitor of ALDH (Supplementary Figures S1 and Figure 2A, 2B, 2D, and 2E). Our data demonstrate that treatment of A375 melanoma cells with 100 μM Lunasin for 24 hours significantly reduced the size of the ALDH^high^ CIC subpopulation (Figure 2C, 2F, and 2G). Our fluorescence analyses for the expression of the ALDH marker corroborated the findings that Lunasin treatment dramatically diminished the ALDH expression levels in both A375 and SKMEL-28 melanoma cells and significantly reduced the ALDH^high^ subpopulation (Figure 2A–2F). To determine if the Lunasin-induced decrease in the ALDH^high^ subpopulation was due to induction of cell death, cell viability assays (Annexin V/PI staining) were performed. Consistent with our initial MTS studies, we failed to detect a significant reduction of cell viability in A375 and SKMEL-28 cells with Lunasin treatment at 100 μM (Supplementary Figure S2), suggesting that Lunasin affects the phenotype of the ALDH^high^ melanoma CICs rather than inducing cell death.

### Lunasin suppresses the functional properties of melanoma CICs

**Figure 3:**
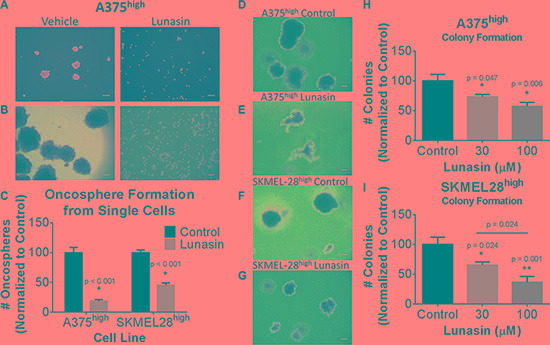
Lunasin reduced self-renewal capacity and oncosphere formation of CICs ALDH^high^ populations of melanoma cells were plated in low adherent culture for 21 d and treated with vehicle or Lunasin twice weekly. Representative images taken at 7 d (**A**) and 21 d (**B**) illustrate the ability of Lunasin to inhibit sphere formation of A375 ALDH^high^ melanoma cells. Lunasin treatment decreased sphere formation by 81% and 55% in A375 ALDH^high^ and SKMEL-28 ALDH^high^ cell lines, respectively (**C**). ALDH^high^ melanoma cells showed increased sensitivity to Lunasin versus their parental counterparts when treated in soft agar. Lunasin-treated ALDH^high^ cells derived from A375 and SKMEL-28 lines exhibited a decreased ability to form colonies in soft agar. Representative images for A375 and SKMEL-28 colonies treated with vehicle (**D**, **F**) or Lunasin (**E**, **G**) illustrate the morphological differences between treatment groups. Both the number and size of colonies formed by Lunasin-treated cells were decreased in size and density in ALDH^high^ fractions of A375 (**H**) and SKMEL-28 (**I**) cell lines. Significance (*p* < 0.05) was determined from three independent experiments and assessed by student's *t-test*. White scale bars shown on images represent 100 μm. All representative images were taken at 40× magnification. Error bars displayed on graphs represent mean ± S.D.

CICs are characterized by having stem-cell-like properties, including the ability to self-renew. Oncosphere formation assays have been widely used to measure the functional activity of CICs, and previous studies have indicated that the ALDH^high^ melanoma cells represent the CIC-enriched compartment [13]. To determine the effects of Lunasin on melanoma CIC functional properties, we performed clonogenic and sphere formation assays with isolated ALDH^high^ A375 and SKMEL-28 melanoma cells. We first established stringent assay conditions in which spheres (floating) and colonies (formed in soft agar) are all of clonal origin. Under these conditions, treatment with 100 μM Lunasin significantly inhibited sphere establishment in ALDH^high^ A375 melanoma cells and in ALDH^high^ SKMEL-28 melanoma cells (Figure 3A–3C). Similarly, Lunasin-treatment at both 30 μM and 100 μM drug doses significantly affected colony forming ability in isolated ALDH^high^ A375 melanoma cells (Figure 3D, 3E, and 3H) as well as in ALDH^high^ SKMEL-28 cells (Figure 3F, 3G, and 3I). In addition to reducing the number of colonies formed, Lunasin also caused a significant reduction in the size of the colonies that did form (Figure 3D–3G). Collectively, these results indicate that treatment with Lunasin negatively regulates melanoma CIC functional properties *in vitro*. In these assays, we demonstrated Lunasin functionally represses the clonogenic ability of melanoma CICs by inhibiting their potential to form oncospheres in non-adherent conditions as well as self-renewal capacity when suspended in soft agar.

### Lunasin limits *in vivo* growth of tumors initiated by melanoma CIC-enriched ALDH^high^ cells

**Figure 4:**
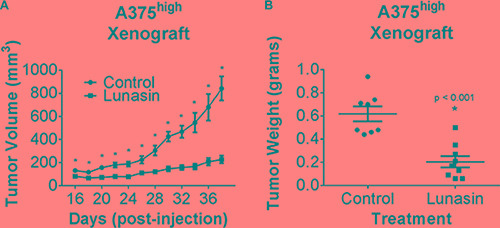
Lunasin inhibited CIC tumorigenesis *in vivo* Athymic nude mice were injected s.c. with 1 × 10^4^ A375 ALDH^high^ cells and subsequently dosed with either 30 mg/kg of Lunasin or vehicle every day for 38 d. Upon endpoint, average tumor volumes in Lunasin (*n* = 9) treated mice were significantly lower compared to mean tumor volume in vehicle (*n* = 8) treated mice (**A**). Upon resection of tumor tissues, wet tumor weights were determined and found to be reduced in Lunasin-treated mice (**B**). Statistical significance between mean tumor volumes of control and treatment groups was determined using GraphPad ANOVA analysis tool (*p* < 0.05). Statistically significant differences in tumor volumes and wet tumor weights were determined by student's *t*-tests, and are denoted by an asterisk (*). Error bars represent mean ± S.E.M.

We further tested if the anti-CIC activity of Lunasin in melanoma *in vitro* translated into better therapeutic efficacy against *in vivo* melanoma CIC-initiated tumor growth. ALDH^high^ A375 cells isolated from the parental line were subcutaneously injected into the dorsal side of nude mice and subsequently treated via the intraperitoneal route with 30 mg/kg of Lunasin or vehicle control as previously described. All mice eventually formed palpable tumors regardless of treatment group; however, a significant reduction in the tumor growth rate was observed in the Lunasin-treated group when compared to that in vehicle treated mice (Figure 4A and 4B). Notably, tumor volumes in Lunasin-treated mice were reduced by 73% (*p < 0.001*; Figure 4A). Also, wet weights of tumors isolated from Lunasin-treated groups were reduced by 67% when compared to tumor weights from vehicle-treated mice (*p < 0.001*; Figure 4B). Additionally, we observed a significant lag time until palpable tumors (> 50 mm^3^) were formed in the Lunasin-treated group when compared to vehicle-treated animals.

We also evaluated if any toxicological effects are associated with daily Lunasin treatment for 34 days. CBC analysis showed no significant difference in blood cell counts between vehicle-treated and Lunasin-treated mice. Additionally, mice receiving Lunasin treatment did not display significantly altered liver enzymes or creatinine levels when compared to the control group; however, 20% lower BUN levels were observed in Lunasin-treated mice (Supplementary Figure S3). These studies indicate that melanoma CICs were very sensitive to Lunasin treatment *in vivo*, and that this treatment did not have any toxic effects.

### Lunasin induces expression of differentiation markers in melanoma CICs

**Figure 5:**
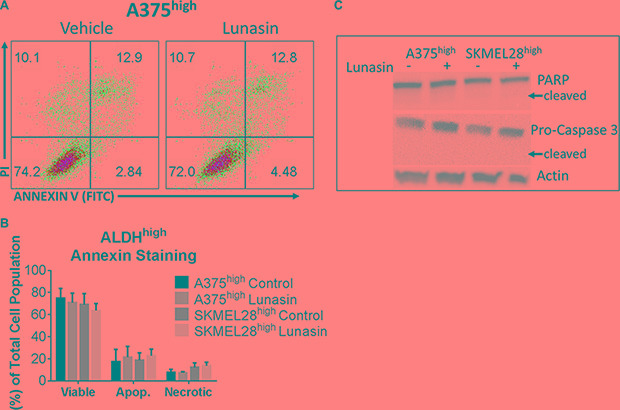
Lunasin did not induce an apoptotic response in ALDH^high^ melanoma cells Annexin V binding assays were used to assess apoptotic populations of melanoma cells sorted for high ALDH activity and subsequently treated with Lunasin or vehicle for 24 h. Representative flow cytometry plots for A375 ALDH^high^ cells in each treatment group are shown (**A**). No significant decrease in cell viability of ALDH^high^ subpopulations derived from A375 or SKMEL-28 cells were observed when stained with Annexin V/PI. Additionally, we did not observe a significant change in apoptotic or necrotic populations after Lunasin treatment (**B**). We further confirmed that Lunasin did not induce apoptosis by immunoblot analysis for the apoptotic markers Caspase-3 and PARP (**C**). Actin was used as a reference protein. Significance was determined by student's *t-test* from three independent experiments with a *p-value* of < 0.05 representing statistical significance. Error bars show mean ± S.D.

**Figure 6:**
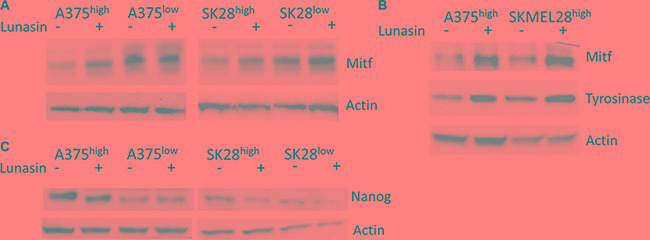
Lunasin modulated expression of melanocyte differentiation and stem-cell-associated markers Sorted subpopulations of ALDH^low^ and ALDH^high^ cells displayed differential expression of the melanocyte-associated transcription factor MITF, with ALDH^low^ cells expressing higher levels of MITF than ALDH^high^ populations in both A375 and SKMEL-28 cell lines (**A**). MITF and the downstream melanocyte differentiation marker Tyrosinase were strongly induced in ALDH^high^ cells treated with Lunasin for 24 h (**B**). Immunoblot analysis revealed that NANOG, a stem-cell-associated marker, was significantly repressed in Lunasin-treated ALDH^high^ samples (**C**). Actin was used as a reference.

To test if the Lunasin-induced decrease in CIC function is due to induction of apoptotic cell death in the isolated CICs, we performed cell viability and apoptosis assays (Annexin V/PI staining) on isolated ALDH^high^ cells following Lunasin treatment. Interestingly, we failed to detect any significant reduction in cell viability with Lunasin treatment in ALDH^high^ CICs obtained from either A375 or SKMEL-28 cell lines when compared to that in vehicle-treated ALDH^high^ CICs (Figure 5A and 5B). Consistent with these results, we did not observe any differences in the protein expression of apoptotic signaling mediators, PARP and Caspase-3 in vehicle and Lunasin-treated ALDH^high^ CICs from both A375 and SKMEL-28 melanoma cell lines (Figure 5C). Recent studies established that reduced expression of MITF yielded G1-arrested cells with an invasive stem-cell-like phenotype, whereas high MITF expression generated either proliferating cells or cells with a differentiated pigment-producing phenotype depending on the status of MITF's post-translational modifications [51, 52]. Other studies provide supportive evidence to this claim and show that TGF-b signaling can mediate MITF expression, which is critical for the generation and maintenance of melanoma stem cells [53]. In accordance with these reports, we examined MITF levels and observed significantly lower MITF expression in CIC-enriched ALDH^high^ A375 and SKMEL-28 cells in comparison to MITF expression in ALDH^low^ A375 and SKMEL-28 cells (Figure 6A).

We next asked whether Lunasin treatment can modulate MITF expression in CICs and thereby, trigger a phenotypic switch in CICs that will ultimately drive the Lunasin-treated melanoma CICs towards differentiation. To test this hypothesis, ALDH^high^ cells isolated from A375 and SKMEL-28 melanoma lines were cultured in the presence or absence of 100 μM Lunasin for 24 hours under serum-free, non-adherent culture conditions. Lunasin-treated and vehicle-treated cultures were then analyzed for MITF protein expression as well as for the expression of its downstream differentiation-associated protein, Tyrosinase. We found that Lunasin treatment significantly increased the expression of MITF and Tyrosinase proteins in A375- and SKMEL-28-derived ALDH^high^ cells compared to vehicle-treated controls (Figure 6B). Additionally, Lunasin-treated ALDH^high^ cells from both A375 and SKMEL-28 melanoma lines showed reduced expression of the stem-cell-associated transcription factor, NANOG (Figure 6C). Taken together, these experiments provide substantial experimental evidence to support the notion that Lunasin treatment negatively influenced melanoma CICs’ tumorigenicity and self-renewal abilities by regulating MITF signaling, one of the key drivers in inducing a differentiated phenotype in melanoma CICs, as well as suppressing levels of the key stemness factor NANOG.

## DISCUSSION

Consumption of large amounts of soy-derived foods is associated with a lower risk of a number of chronic diseases including cancer [54–56]. The anti-cancer effects of soy components have been attributed to secondary metabolites such as isoflavones and specific protein fractions [57, 58]; however, no epidemiological evidence directly correlating soy consumption with decreased melanomagenesis has come to light. Lunasin, a peptide present in crude soy protein, has been proposed to be an important chemoprevention agent in soy [30]. Lunasin is a 43–44-amino acid polypeptide [24, 59] that is encoded within the soybean GM2S-1 gene. The 22-amino acid N-terminal sequence (with no known function) of Lunasin is followed by a putative helix domain proposed to target Lunasin to chromatin, and the C-terminal end that includes a RGD cell adhesion motif followed by a poly-aspartic acid tail [24, 25]. Lunasin's potential chemopreventive activity has been established by studies showing that Lunasin prevents cellular transformation by chemical carcinogens and viral oncogenes [30]. Recent studies have shown that Lunasin can inhibit the *in vitro* and *in vivo* growth of breast [34, 60], leukemia [61], colon [31, 61], and lung cancers [35]. Here, our findings reveal for the first time that Lunasin has significant therapeutic effects against melanoma in both non-adherent *in vitro* assays and *in vivo* xenograft studies.

The ALDH^high^ melanoma cancer cell subpopulation has been reported to harbor the tumor-initiating and metastatic cells that express several self-renewal stemness genes including *NANOG* [62, 63]. A recent study has shown that siRNA-mediated knockdown of ALDH in melanoma cells inhibited *in vivo* tumor development and metastatic properties [64]. Mechanistically, the ALDH^high^ melanoma cells have been shown to possess higher tumorigenic, invasive, and self-renewal capacities than ALDH^low^ cells and thus, can serve as a potential therapeutic target [13, 62, 65]. These studies implicate high ALDH activity as a relevant biomarker for identifying melanoma CICs, and the significant inhibition of ALDH activity that we observed with Lunasin phenocopies the anti-melanoma/anti-CIC effects observed in cells silenced for ALDH. Using this surrogate assay for CIC identification, we demonstrated that sorted cell populations based on the ALDH biomarker were sensitive to Lunasin treatment in non-adherent melanosphere formation assays as well as colony formation in soft agar. Our studies further showed that Lunasin functionally blocked the self-renewal capacity of isolated melanoma CICs. The activity of Lunasin both *in vitro* and *in vivo* on melanoma CICs suggests the intriguing possibility that Lunasin can target these quiescent and drug-resistant cells and is consistent with a recently published study on colon cancer [33]. These studies demonstrated that putative CICs isolated based on the expression of the surface markers CD133 and CD44 were sensitive to Lunasin and that this effect likely involves modulation of the PTEN/PI3K/FASN axis [33]. Taken in combination with our results, these data support a novel hypothesis that Lunasin and other soy derivatives have the ability to decrease cancer stem cell populations. An important and intriguing aspect of our study is that the anti-cancer effects of Lunasin were enduring *in vivo*. Despite somewhat modest effects *in vitro*, we observed a highly significant effect on tumor growth when cells were transplanted into immunodeficient mice. Lunasin-treated mice had a significantly reduced tumor burden in both parental (46%) and ALDH^high^ (73%) A375 cells when compared to their vehicle-treated counterparts.

With regard to potential mechanisms of action, Lunasin contains a RGD domain and has been shown to bind specific integrins that recognize this cell adhesion motif [37]. Integrins are heterodimeric cell surface proteins that play critical roles in adhesion to the extracellular matrix, transmitting extracellular signals that affect cell migration and the regulation of signaling pathways involved in cell survival and proliferation. Recent studies [26, 32, 35, 66] strongly suggest that Lunasin bound to integrins containing combinations of the α5, αv,β1, and β3 subunits and modulated the Integrin-Linked Kinase (ILK) and Focal Adhesion Kinase (FAK) signaling pathways. Additionally, it is becoming clear that there is a strong linkage of integrin-matrix interactions to cancer cell initiation and progression (reviewed in [45]), including the maintenance and survival of CICs through integrin-FAK signaling (reviewed in [67]). Interestingly, our current data indicate that isolated ALDH^high^ melanoma cells are more sensitive to Lunasin than the unsorted cell fractions. One possible explanation for this differential sensitivity is that when compared to the bulk of tumor cells, the ALDH^high^ subpopulation of melanoma cells exhibit altered integrin expression profiles. It is also possible that the ALDH^high^ melanoma CICs rely more heavily and specifically on ‘outside-in’ signal transduction mechanisms mediated via integrin networks compared to ALDH^low^ cells. Such differential integrin signaling in CICs could confer an increased sensitivity to Lunasin. In fact, it has been reported that metastatic melanomas, compared to primary melanomas, favor the expression of particular integrins, including integrin α_v_β_3_ [68], a known Lunasin target [35]. Differential expression of integrins in CICs is not only restricted to melanoma but have been reported in CICs from other cancers including prostate [69, 70], breast [71], and neuroglia cancers [47]. Given the strong interaction between Lunasin and integrins [26, 35, 37], it is tempting to speculate that Lunasin specifically targets CICs by modulating integrin signaling circuits that are differentially expressed in melanoma CICs. Although the mode of action of Lunasin's anti-CIC activity remains to be clearly defined, our future studies will focus on identifying the specific integrin-mediated signaling modules required for Lunasin efficacy against melanoma CICs.

MITF, commonly referred to as a “master controller” gene for melanocyte development, strictly regulates melanocyte proliferation and differentiation [72]. Recent studies have identified the existence of slow-cycling, low MITF-expressing CICs in melanoma cell populations with intrinsic chemoresistant and tumorigenic phenotypes [73]. Additionally, this subpopulation of melanoma cells expressed high levels of the stem-cell-associated markers Oct-4 and NANOG [73]. One of the most significant findings from our study is that treatment of melanoma ALDH^high^ CICs with Lunasin induced a more differentiated phenotype by increasing the expression of MITF as well as the expression of the downstream melanocyte differentiation marker, Tyrosinase, an enzyme directly involved in melanin synthesis. Concomitant with the Lunasin-induced phenotypic shift, we observed a significant reduction in the expression of NANOG, a transcription factor implicated in migration, invasion, self-renewal, and dedifferentiation of melanoma cells [74–76]. This represents a novel activity for Lunasin that has not been reported in any cancer model to date. The specific regulatory pathways affected by Lunasin to effect MITF and NANOG expression in melanoma ALDH^high^ cells remain to be identified. As previously discussed, Lunasin has been shown to inhibit histone acetylation and function as an integrin-signaling antagonist via interactions with αv-containing integrins [26, 35]. Based on studies in other cancer cell types, specific pathways that may be involved in the observed effects of Lunasin on melanoma CICs include suppression of Akt, ERK1/2, and NF-κB and activation of the tumor suppressor PTEN [32, 33, 35, 60]. The ability of Lunasin to suppress ERK1/2 expression is particularly intriguing given reports that activation of ERK1/2 can upregulate ubiquitin-dependent degradation of MITF [77].

**Figure 7:**
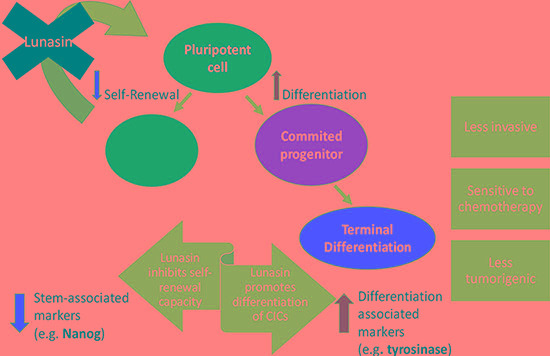
Proposed mechanism for Lunasin's activity in melanoma CICs This diagram depicts the observed effects and possible therapeutic advantage of Lunasin treatment in cases of malignant melanoma. Lunasin decreased the stem-cell-like properties of ALDH^high^ CICs isolated from A375 and SKMEL-28 cell lines while concurrently decreasing the stem-cell-associated marker NANOG and inducing expression of melanocyte differentiation markers MITF and Tyrosinase. By effectively reducing this stem-cell-like compartment, Lunasin may alleviate patient relapse caused by the subpopulation of cells with intrinsic metastatic potential and chemoresistance.

Taken together, results from our present study and previously published data support the model of the potential therapeutic benefits of Lunasin in melanoma depicted in Figure 7. By effectively reducing pools of CICs by driving a movement of cells out of the cancer stem-cell-like compartment (i.e. ALDH^high^) and into a more differentiated phenotype (i.e. ALDH^low^), Lunasin may alleviate patient relapse by diminishing pools of cells with the intrinsic abilities generally conserved in hematopoietic stem cells. By blocking self-renewal and subsequent expansion of the CIC compartment, Lunasin may ultimately prove to be an indispensable tool in combating populations of cells with high invasive potential and chemoresistant characteristics.

## MATERIALS AND METHODS

### Isolation and purification of lunasin

Lunasin was isolated from soybean white flake (provided by Owensboro Grain Company, Owensboro, KY), a product resulting from the flaking and defatting of soybeans via hexane extraction. The extraction and purification was performed by Kentucky BioProcessing (Owensboro, KY) as previously described [24]. Sodium dodecyl sulfate polyacrylamide gel electrophoresis (SDS-PAGE) analysis indicated that these Lunasin preparations had > 99% purity (Supplementary Figure S4). Purified Lunasin was suspended in sterile 50 mM sodium phosphate buffer, pH 7.4 (PB) and stored at 4°C.

### Cell culture and reagents

SKMEL-28 and A375 cell lines were obtained from American Type Culture Collection (Rockville, MD), and further authenticated by short tandem repeat profiling (Promega). Cells were monitored for mycoplasma contamination every 6 months. All cell lines were grown in Dulbeccos Modified Eagles Medium (DMEM) supplemented with 10% Fetal Bovine Serum (FBS), Penicillin (100 U/mL), and Streptomycin (100 μg/mL). Cells were incubated at 37°C at 5% CO_2_ and sub-cultured every 72 hours. ALDH^high^ cells were isolated by fluorescence-activated cell sorting (FACS) and were grown in DMEM/F-12 serum-free media containing 1 × N-2 Supplement (Gibco) 10 ng/mL basic fibroblast growth factor (Gibco), and 10 ng/mL epidermal growth factor (Gibco). For soft agar assays, DMEM media (Invitrogen) powder was reconstituted in ultrapure water (500 mL) and supplemented with 20% FBS, Penicillin (200 U/mL), and Streptomycin(200 μg/mL).

### Soft agar colony forming assay

A lower, cell-free layer of 0.5% Bacto agar and cell culture media (1:1 suspension) was plated in 6-well tissue culture plates and allowed to solidify at room temperature in a laminar flow cabinet. An upper layer of 0.35% agar and culture media (1:1) containing 1000 melanoma cells plus Lunasin or vehicle control was plated over the solid lower layer. Plates were incubated at 37°C and 5% CO_2_ for 10–18 days until colonies grew to approximately 100 μm in diameter. After seeding, plates were fed with culture media containing vehicle (PB) or Lunasin twice weekly. Plates were stained with crystal violet solution (0.005%; Sigma-Aldrich), photographed, and scanned (1000 dpi; EPSO Expression 1680 scanner). Average colony size and total colony area for each sample were analyzed using Image-J software (National Institutes of Health).

### ALDH assays

The ALDH positive population was identified using a commercial kit (ALDEFLUOR™, Stem Cell Technologies) according to manufacturer's directions. A375 and SKMEL-28 cells were grown to approximately 80% confluence in DMEM cell culture medium and treated with Lunasin for 24 h. Cells (1 × 10^6^ cells/mL) were washed and resuspended in ALDEFLUOR™ Assay Buffer. ALDEFLUOR™ reagent (5 μL/mL) was added to the cell suspension. The sample was mixed, and a portion was added to a fresh tube containing the N,N-diethylaminobenzaldehyde (DEAB) inhibitor. Another portion was placed in a fresh tube for staining with 10 μg/mL propidium iodide. Samples were incubated at 37°C for 45 minutes and mixed occasionally by inversion. Flow cytometry was performed using FACSCalibur (BD Biosciences).

### Annexin V binding assays

Melanoma cells (2.5 × 10^5^ cells) were seeded in a T-25 culture flask and allowed to adhere for 4 hours. Culture media was replaced and amended with media containing 100 μM Lunasin or vehicle for 24 h. Cells were harvested using enzyme-free trypsin (TrypLE, Life Technologies), counted, and subjected to labeling. For identifying ALDH^high^ cells, melanoma cells were assayed with ALDH as described above. ALDH^high^ and ALDH^low^ melanoma cells were sorted using a MoFlo cell sorter (Dako Cytomation). Sorted ALDH^high^ cells (1 × 10^3^ cells/mL) were plated in low-attachment 6-well plates (Corning) in DMEM/F-12 serum-free media. Cells were either treated with 100 μM Lunasin or vehicle and labelled with Annexin V/PI. Labelled cells were analyzed by flow cytometry using FACSCalibur (BD Biosciences).

### Melanoma oncosphere culture

A375 and SKMEL-28 melanoma cells were assayed for ALDH as described above, and the ALDH^high^ cells were sorted using a MoFlo flow cytometer (Dako Cytomation). Gates were set based upon DEAB controls for each cell line and reflected at least a one-log shift between negatively and positively stained cells. A typical staining profile using the MoFlo cell sorter for SKMEL-28 cells is shown in Supplementary Figure S1. The average ALDH^high^ subpopulation was 38% and 17% of the total cells for A375 and SKMEL-28, respectively. Sorted cells were cultured in low-adherent T-25 flasks (Corning) in DMEM/F-12 serum-free media at a density of 1 × 10^3^ cells/mL. Cultures were grown for up to 21 days and treated with fresh media containing either 100 μM Lunasin or vehicle twice per week. Oncospheres (> 100 μm) were harvested and passed through a 70 μm nylon filter (BD Biosciences) to remove single cells and small cell clumps. Spheres were imaged and analyzed using Image-J software [78].

### Xenograft experiments

Male athymic nude mice (Jackson #002019) were used at 6–8 weeks of age. All mice were handled in accordance with the Association for Assessment and Accreditation of Laboratory Animals Care international guidelines with the approval of the appropriate Institutional Animal Care and Use Committees at the University of Louisville (protocol #12091) and Indiana University, Bloomington (Protocol # 14–019–4). Mice were injected s.c. with 2.5 × 10^6^ A375 cells in phosphate buffered saline (PBS; 100 μL). Mice received daily i.p. injections of either Lunasin (30 mg/kg) or vehicle (PB) starting the same day that cells were implanted. To test the *in vivo* properties of CICs, ALDH^high^ A375 cells were sorted as described above. ALDH^high^ cells (1 × 10^4^) were mixed at a ratio of 1:1 with Matrigel (growth factor reduced, without phenol red; BD Biosciences) and 100 μL of this matrigel-cell suspension was implanted s.c. on the dorsal side of the athymic nude mice. Tumor size was monitored thrice weekly until animals were sacrificed due to tumor burden. Tumor volume [V = L × W2 × (*π*/6)] was determined by measuring the greatest linear dimensions in length (L) and width (W).

### Immunoblot analysis

Cultured cells were treated with PB or Lunasin (100 μM) for 24 hours. Cells were harvested and re-suspended in RIPA buffer (Sigma-Aldrich). Protein concentrations of cell lysates were determined by a bicinchoninic acid assay (Thermo Fisher Scientific) and 20–40 μg of total protein was loaded per lane on 10% gels (BioRad), subjected to SDS-PAGE, and transferred to a PVDF membrane (EMD Millipore). Lysates were probed with antibodies that recognize human MITF (Cell Signaling #12590), Tyrosinase (EMD Millipore #05–647), poly ADP ribose polymerase (PARP, Santa Cruz #sc-7150), Caspase-3 (Santa Cruz #sc-56055), NANOG (EMD Millipore #MABD24), and β-Actin (Santa Cruz #sc-47778). Densitometry and image analysis were performed using a ChemiDoc station equipped with ImageLab software (BioRad).

### Toxicological analysis

Whole blood was drawn from athymic nude mice by cardiac puncture immediately following CO_2_ asphyxiation and collected in serum separator tubes (BD Biosciences) or EDTA coated collection tubes (BD Biosciences). 25 μL of whole blood was collected in EDTA coated tubes and analyzed by the Research Resource Center (RRC) facility at the University of Louisville for complete blood count (CBC) analysis. After 1 hour post-collection, whole blood samples collected in serum separator tubes were centrifuged for 10 minutes at 10,000 g. 250 μL of serum was removed from each sample, collected in a 1.5 mL eppendorf tube, and analyzed by the RRC facility for non-steroidal anti-inflammatory drug (NSAID) toxicological analysis. Liver damage was assessed by levels of alanine aminotransferase (ALT), aspartate aminotransferase (AST), and alkaline phosphatase (ALKP). Kidney damage was assessed by levels of blood urea nitrogen (BUN) and creatinine (CREA).

### Statistical analysis

GraphPad Prism 5.0 software (GraphPad Prism Software, Inc., La Jolla, CA) was used for all statistical analyses. For all *in vitro* studies, two-group comparisons between control and test samples (groups compared are indicated in the respective Figures) were done by two-tailed student's *t*-tests and represent data from three independent experiments. For the *in vivo* tumor measurement studies, group comparisons were done using ANOVA. For all tests, statistical significance was assumed when *p* < 0.05.

## SUPPLEMENTARY MATERIALS FIGURES AND TABLES


