# Supplementary material for: Lunasin is a novel therapeutic agent for targeting melanoma cancer stem cells
Source: Oncotarget. 2016 Aug 23;7(51):84128–41. doi: 10.18632/oncotarget.11554 (PMC5356649; doi:10.18632/oncotarget.11554)
Supplement: Supplementary file 1 [file oncotarget-07-84128-s001.pdf]

Lunasin is a novel therapeutic agent for targeting melanoma cancer stem cells

Supplementary Materials

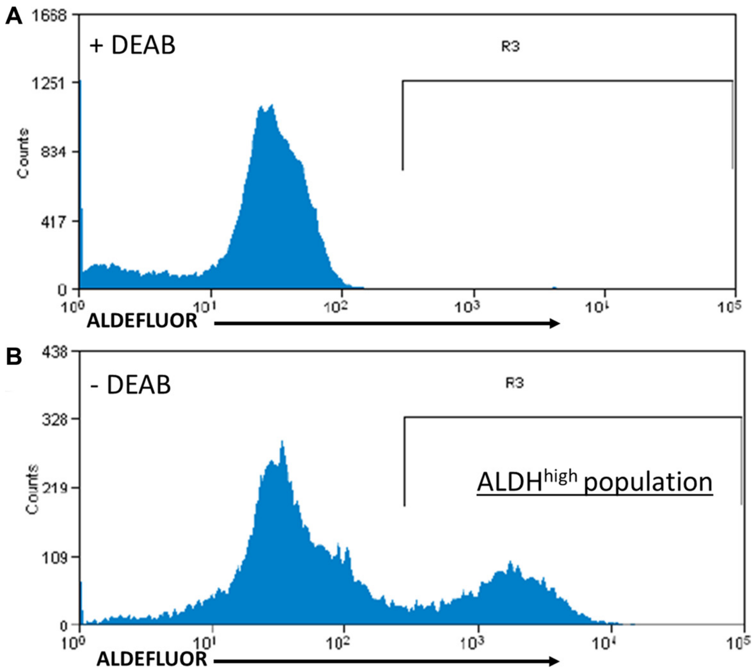

Supplementary Figure S1: SKMEL-28 cells stained with ALDEFLUOR reagent +/- DEAB. SKMEL-28 melanoma cells were grown in adherent culture, harvested, and stained for ALDH activity. This histogram represents a typical ALDH labeling profile for the SKMEL-28 cell line, and shows subpopulations both negative (**A**) and positive (**B**) for ALDH activity.

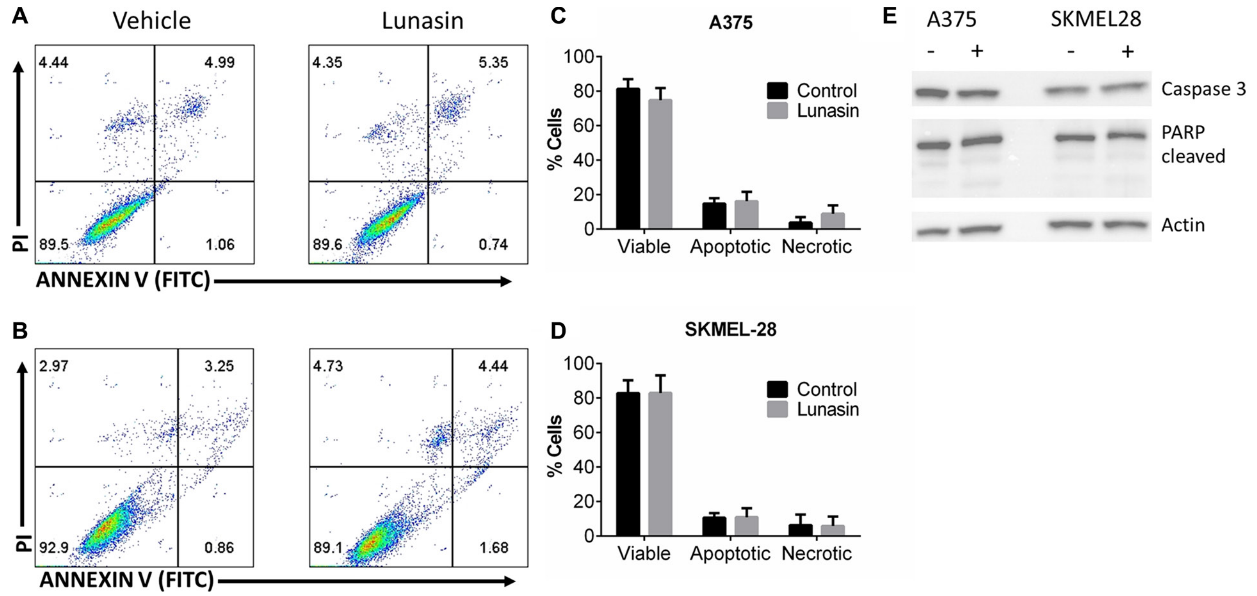

Supplementary Figure S2: Lunasin did not induce apoptosis in parental melanoma cell lines. A375 (**A**, **C**) and SKMEL-28 (**B**, **D**) cell lines were treated with 100 μM Lunasin for 24 h, and subsequently analyzed for apoptosis. No significant induction of apoptosis or necrosis was observed with Lunasin treatments. These results were corroborated by findings that Lunasin did not increase levels of cleaved PARP and Caspase 3 when assessed by immunoblot analysis (**E**).

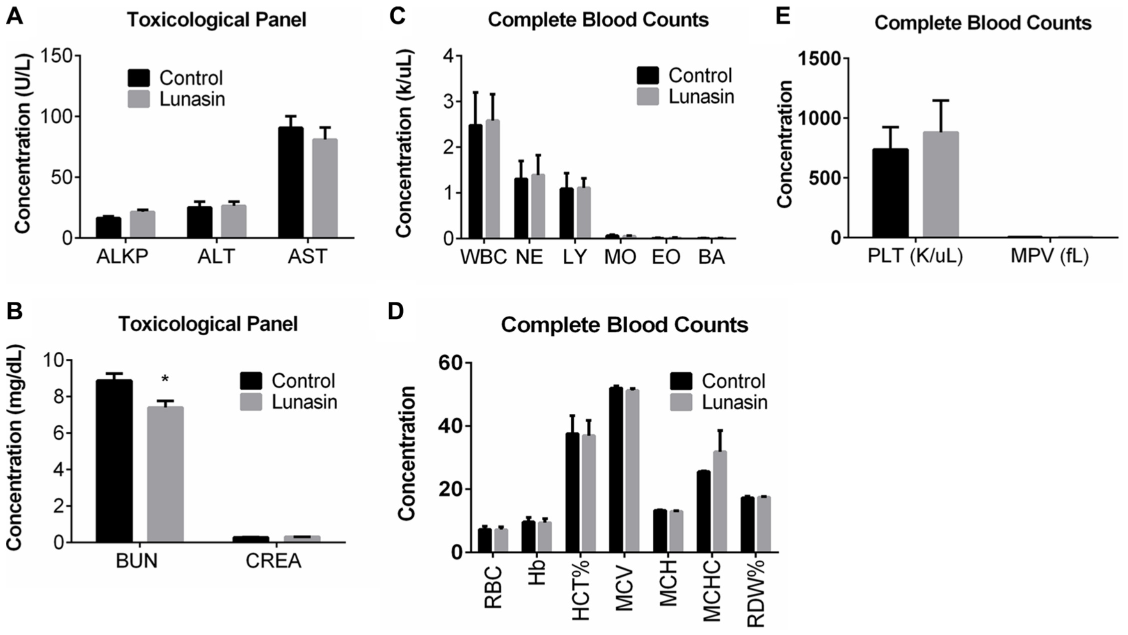

Supplementary Figure S3: Long-term Lunasin treatment did not induce toxic side effects. Nude mice were treated with Lunasin (30 mg/kg) for 34 days. Upon experimental endpoint, blood serum and whole blood fractions were analyzed for liver (**A**), kidney (**B**) and CBC (**C**–**E**) toxicity, respectively. No toxic effects were observed in the Lunasin-treated groups.

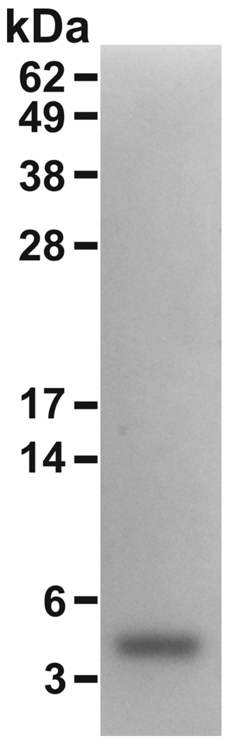

Supplementary Figure S4: SDS-PAGE analysis of lunasin purified from soybean white flake. A total of 5 µg total protein was subjected to SDS-PAGE using a 15% gel (BioRad) followed by staining with Coomassie Brilliant Blue (BioRad). Labels indicate migration of SeeBluee Plus2 (Life Technologies) protein standards
